# Systematic analysis of tumour cell-extracellular matrix adhesion identifies independent prognostic factors in breast cancer

**DOI:** 10.18632/oncotarget.11307

**Published:** 2016-08-17

**Authors:** Jason R. Todd, Karen A. Ryall, Simon Vyse, Jocelyn P. Wong, Rachael C. Natrajan, Yinyin Yuan, Aik-Choon Tan, Paul H. Huang

**Affiliations:** ^1^ Division of Cancer Biology, The Institute of Cancer Research, London, UK; ^2^ Department of Medicine, Translational Bioinformatics and Cancer Systems Biology Laboratory, Division of Medical Oncology, University of Colorado Anschutz Medical Campus, Aurora, CO, USA; ^3^ The Breast Cancer Now Toby Robins Research Centre, Division of Breast Cancer Research, The Institute of Cancer Research, London, UK; ^4^ Division of Molecular Pathology, The Institute of Cancer Research, London, UK

**Keywords:** cell adhesion, breast cancer, extracellular matrix, laminin, HER2

## Abstract

Tumour cell-extracellular matrix (ECM) interactions are fundamental for discrete steps in breast cancer progression. In particular, cancer cell adhesion to ECM proteins present in the microenvironment is critical for accelerating tumour growth and facilitating metastatic spread. To assess the utility of tumour cell-ECM adhesion as a means for discovering prognostic factors in breast cancer survival, here we perform a systematic phenotypic screen and characterise the adhesion properties of a panel of human HER2 amplified breast cancer cell lines across six ECM proteins commonly deregulated in breast cancer. We determine a gene expression signature that defines a subset of cell lines displaying impaired adhesion to laminin. Cells with impaired laminin adhesion showed an enrichment in genes associated with cell motility and molecular pathways linked to cytokine signalling and inflammation. Evaluation of this gene set in the Molecular Taxonomy of Breast Cancer International Consortium (METABRIC) cohort of 1,964 patients identifies the F12 and STC2 genes as independent prognostic factors for overall survival in breast cancer. Our study demonstrates the potential of *in vitro* cell adhesion screens as a novel approach for identifying prognostic factors for disease outcome.

## INTRODUCTION

The extracellular matrix (ECM) in the mammary gland microenvironment undergoes dynamic remodelling and is deregulated during breast cancer progression [1, 2]. By providing adhesive surfaces that promote adhesome complex formation and the activation of intracellular signalling [3, 4], ECM proteins have been shown to accelerate tumour progression and metastatic disease in breast cancer [5–8]. Degradation of the basement membrane (BM), which is primarily composed of ECM components collagen IV and laminin, is also required for cancer cell invasion into the surrounding blood vessels and lymphatics facilitating metastatic spread [9]. Given the important contributions of the ECM in breast cancer biology, protein expression levels of specific ECM components such as fibronectin and tenascin C are known prognostic markers for poor overall patient survival [10–12]. More broadly, multiple studies demonstrate that gene expression signatures of the breast cancer stroma encompassing ECM proteins are more robust predictors of patient outcome than those of the tumour epithelial [13–16].

While critical for promoting cancer progression, tumour cell-ECM interactions are challenging to study due to the dynamic remodelling of the matrisome *in vivo* [17], the wide array of ECM receptors expressed in cells [18] and the ability of these receptors to synergistically interact in a complex non-intuitive fashion [19, 20]. The multi-factorial nature of these interactions is one reason why targeting ECM receptors, while conceptually attractive, has yet to translate into successful clinical candidates for cancer therapy [21]. To address some of these challenges, large-scale ECM arrays have been developed to examine tumour cell-ECM interactions in a systematic and combinatorial manner [20]. This has led to the characterisation of key adhesive changes associated with metastatic progression in a mouse model of lung adenocarcinoma and provided the first demonstration that *in vitro* cell adhesion screens can be employed as a means to identify clinically meaningful biomarkers such as galectin-3 for tumour, node and metastasis (TMN) staging [20].

The prognostic value of cancer cell adhesion has remained unexplored and we hypothesize that in addition to tumour staging, analysis of tumour cell-ECM interactions may reveal new prognostic factors for disease outcome. To investigate the relationship between tumour cell-ECM interactions and breast cancer survival, in this study we undertook an automated image-based phenotypic screen to assess tumour cell-ECM adhesion profiles in a panel of HER2-amplified (HER2+) breast cancer cell lines. We identified a gene expression signature that defines breast cancer cell lines with impaired adhesion to laminin and correlate these findings with a clinical dataset of 1,964 breast cancer cases to isolate genes that are prognostic for overall survival in patients. Our study demonstrates that cell adhesion screens have the potential to identify novel prognostic factors in breast cancer and is a general approach that can be readily extended to the study of other tumour types.

## RESULTS

### Analysis of tumour cell-ECM interactions reveals a subset of cell lines that display impaired adhesion to laminin

**Figure 1:**
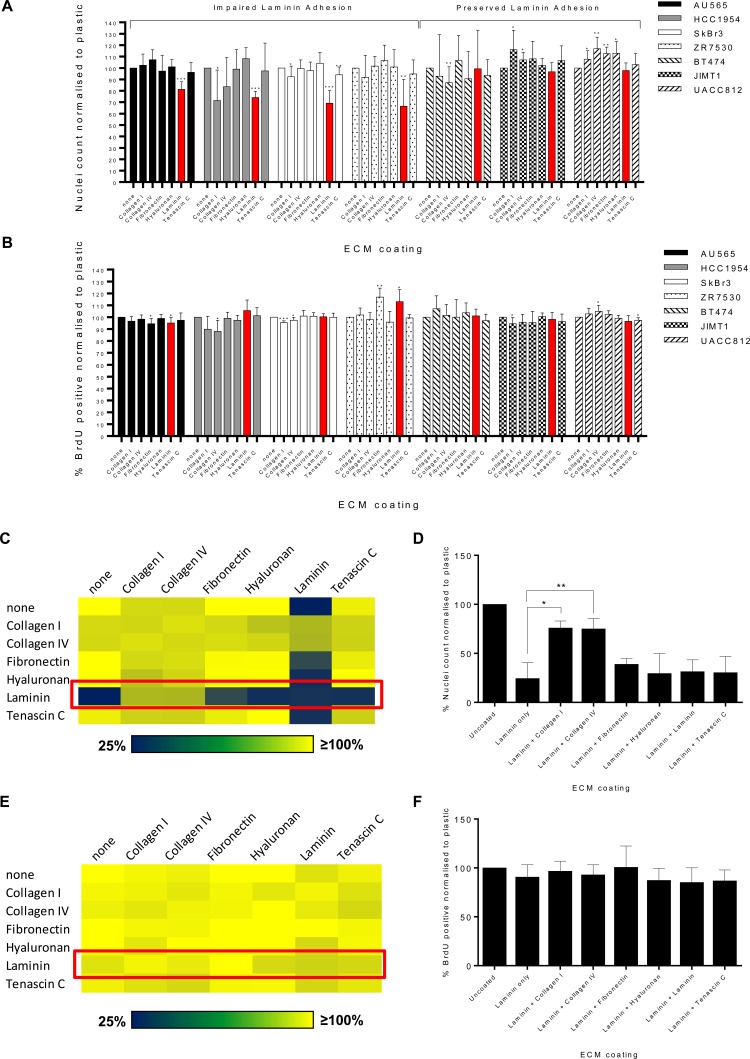
A subset of breast cancer cell lines display impaired adhesion of laminin Bar plots showing percentage **A.** DAPI stained nuclei counts or **B.** BrdU incorporation in a panel of seven HER2+ breast cancer cell lines on six ECM substrates. Data is normalised to the plastic control (*n* = 7 or 8). Statistical analysis comparing ECM substrate versus plastic control was performed by paired Student's t test where *p < 0.05, **p < 0.01 and ***p < 0.001. All values are mean ± SD. **C.** Heatmap of percentage nuclei counts as measured by DAPI staining in the SkBr3 cell line in the presence of pairwise combinations of ECM substrates. Red box highlights combination of laminin with other ECM substrates. **D.** Bar plots of percentage nuclei counts in SkBr3 cells under conditions of pairwise combinations of laminin and an additional ECM substrate. Data is normalised to uncoated plastic control (*n* = 3). Statistical analysis of combined ECM substrate versus laminin only was performed by paired Student's t test where *p < 0.05 and **p < 0.01. All values are mean ± SD. **E.** Heatmap of percentage BrdU incorporation in the SkBr3 cell line in the presence of pairwise combinations of ECM substrates. Red box highlights combination of laminin with other ECM substrates. **F.** Bar plots of percentage BrdU incorporation in SkBr3 cells under conditions of pairwise combinations of laminin and an additional ECM substrate. Data is normalised to uncoated plastic control (*n* = 3). All values are mean ± SD.

To systematically characterise tumour cell-ECM interactions in breast cancer, we utilised a panel of seven well-annotated HER2+ breast cancer cell lines [22, 23]. HER2 overexpression or amplification is present in ~20% of breast cancers and is associated with poor prognosis and aggressive disease [24]. We employed automated image-based phenotypic screens to evaluate the manner in which HER2+ cells adhere to ECM molecules. 96-well plates were pre-coated with six ECM components that are commonly deregulated in breast cancer (collagen I, collagen IV, fibronectin, hyaluronan, laminin, tenascin C) with uncoated plastic as a negative control [25]. Cells were subsequently seeded and screened for cell adhesion at 72 hours using DAPI as a nuclei stain (Figure 1).

Mining the phenotypic dataset, we identified a subset of cell lines (AU565, HCC1954, SkBr3 and ZR75.30) that displayed significantly impaired cell adhesion when seeded on laminin (Figure 1A). The remaining three cell lines BT474, JIMT1 and UACC812 showed normal adhesion to laminin versus plastic. To demonstrate that the observed decrease in nuclei counts is not the result of reduced cell proliferation in response to ECM exposure, we assessed BrdU incorporation in the cell line panel on the 6 ECM substrates (Figure 1B). ECM substrates did not reduce cell proliferation across the panel of cell lines while increased proliferation was only found in the ZR75.30 cells when exposed to fibronectin and laminin. These data confirm that the observed decrease in nuclei count in AU565, HCC1954, SkBr3 and ZR75.30 cell lines is due to a reduction in cell adhesion on laminin rather than a decrease in proliferative rate.

### Combinations of collagens with laminin rescues SkBr3 cell adhesion

A previous unbiased screen of lung adenocarcinoma cell-ECM adhesion showed that distinct combinations of ECM substrates results in unanticipated additive, synergistic or antagonistic effects on cell adhesion [20]. We sought to determine if similar effects were present in breast cancer, using the laminin adhesion impaired SkBr3 cell line as a model. These cells were subjected to pair-wise combinations of the 6 ECM substrates and plastic for 72 hours prior to assessment for nuclei counts. This screen revealed that both collagen I and collagen IV were able to rescue the impaired tumour cell adhesion when combined with laminin (Figure 1C & 1D). Assessment of BrdU incorporation in the combination ECM format showed that there were no significant differences in proliferation across all laminin-ECM combinations, indicating that the observed rescue of cell adhesion in the presence of collagen was independent of cell proliferation (Figure 1E & 1F).

Analysis of publically available transcriptional profiles of the seven cell lines finds that cell adhesion profiles on single ECMs had no correlation with the mRNA expression levels of their cognate laminin or collagen receptors such as the integrins (Table 1) [26, 27]. The exception is the positive correlation between collagen IV and Discoidin Domain Receptor 1 (DDR1) (correlation coefficient *R* = 0.88, *p* = 0.01). It should be noted that the data in Table 1 reflects the gene expression levels of these receptors at steady state in the breast cancer cell line panel and not in response to ECM exposure. Importantly we show by 2-way clustering of mRNA levels of the laminin-binding integrins that there is no obvious correlation between laminin adhesion and integrin mRNA levels (Supplemental Figure 1A). For instance, HCC1954 has high levels of laminin-binding integrin mRNA levels but displays impaired adhesion. In addition, SkBr3 and BT474 are part of the same cluster despite displaying opposing laminin adhesion profiles. We have also performed a Student's t-test analysis comparing the laminin-binding integrin levels of both groups of cell lines (impaired laminin adhesion and preserved laminin adhesion) and show that there is no statistical difference in the integrin gene expression levels between the two groups (Supplemental Table 1).

**Table 1:** Correlation of mRNA levels of ECM receptors and cell adhesion

ECM	Receptor	R (Correlation coefficient)	*P*-value
Collagen I	ITGA1	0.46	0.30
	ITGA2	0.22	0.64
	ITGA10	0.08	0.86
	DDR1	0.47	0.29
	DDR2	0.16	0.73
	ITGAX	−0.19	0.68
	ITGB1	0.32	0.48
Collagen IV	ITGA1	0.49	0.26
	ITGA2	0.07	0.87
	ITGA10	0.48	0.27
	ITGB1	0.14	0.76
	DDR1	0.88	0.01*
Hyaluronan	CD44	0.31	0.50
Laminin	ITGA1	0.13	0.78
	ITGA2	0.31	0.49
	ITGA3	0.46	0.30
	ITGA6	0.45	0.30
	ITGA7	0.00	0.99
	ITGA10	−0.21	0.65
	ITGB1	0.62	0.14
	ITGB4	0.10	0.83
Fibronectin	ITGA4	−0.17	0.72
	ITGA5	0.08	0.86
	ITGA8	−0.64	0.12
	ITGAV	−0.52	0.23
	ITGA2B	0.37	0.42
	CD44	0.40	0.37
	ITGB1	0.79	0.04*
	ITGB3	0.11	0.82
	ITGB6	−0.10	0.82
	ITGB7	−0.56	0.19
	ITGB8	−0.20	0.66
Tenascin C	ITGA8	−0.26	0.57
	ITGAV	0.31	0.50
	ITGB1	0.70	0.08
	ITGB3	−0.28	0.54
	ITGB6	0.40	0.37

* Statistical significance of correlation where *p* < 0.05.

To validate the observations identified in the microarray analysis, we performed real-time quantitative PCR measurements (RT-qPCR) of the laminin-binding integrins. Correlation analysis with laminin adhesion (Supplemental Table 2), 2-way clustering (Supplemental Figure 1B) and Student's t-test (Supplemental Table 1) of qPCR data confirms that there is no correlation between laminin adhesion and integrin mRNA levels. This data provides further evidence that gene expression levels of the integrins are not able to classify cells based on their ability to bind laminin. This finding is consistent with the previous tumour cell-ECM screen by Reticker-Flynn et al., which showed that unlike protein level measurements, mRNA levels of adhesion receptors are poor predictors of cell-ECM interactions [20].

### Identification of molecular pathways associated with impaired laminin adhesion

**Figure 2:**
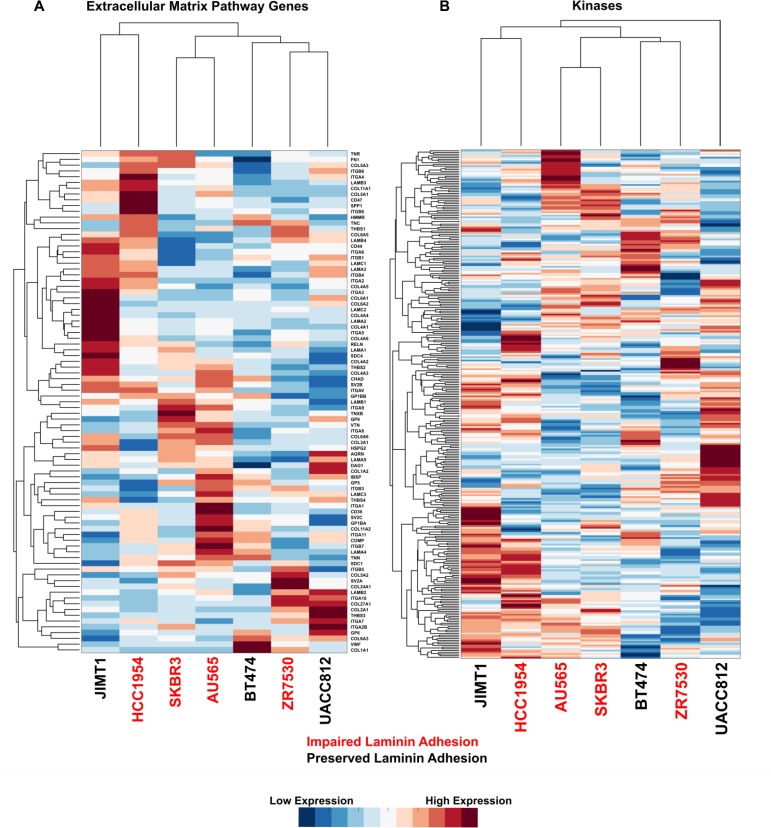
Impaired laminin adhesion cell lines do not cluster based on expression of specific ECM genes or kinases Hierarchical clustering of normalised microarray gene expression data for **A.** KEGG annotated ECM pathway genes and **B.** kinases. Each row was normalised before clustering to give a mean of 0 and a standard deviation of 1. Red indicated higher expression of a gene in the cell line and blue indicates lower expression. Cell lines with impaired laminin adhesion are labelled in red.

To identify molecular pathways associated with impaired laminin adhesion, we mined publically available gene expression data for the cell line panel from the Cancer Cell Line Encyclopaedia (CCLE) [28]. We first examined the ECM pathway genes that are annotated in the Kyoto Encyclopaedia of Genes and Genomes (KEGG) and found that these genes were not able to distinguish cell lines that had impaired laminin adhesion (Figure 2A). Given that the panel of cell lines is driven by the HER2 tyrosine kinase and that the process of cell adhesion is propagated by kinase signalling networks [4], we sought to establish if RNA expression levels of kinases was capable of discriminating the two subsets of cells. An analysis of 298 genes from the kinome showed that kinase profiles have no correlation with the impaired laminin adhesion that was observed in the phenotypic screen (Figure 2B).

**Figure 3:**
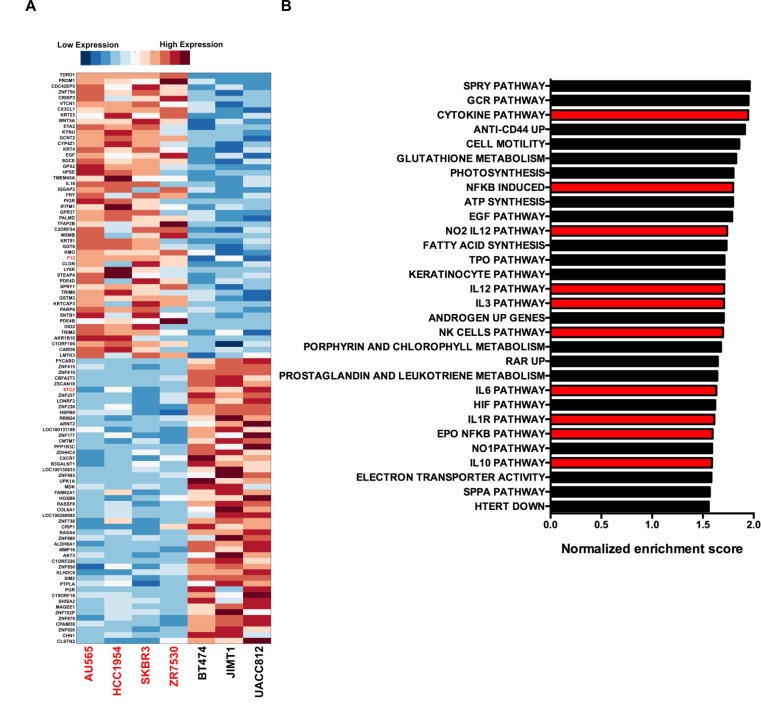
Genes that are correlated with impaired laminin adhesion **A.** Gene expression heatmap for the top 50 genes positively and negatively correlated with the impaired laminin adhesion cell lines. Gene markers were identified using GSEA. Each row of the heatmap was normalized to give a mean of 0 and a standard deviation of 1. Red indicates higher expression of a gene and blue indicates lower expression. FDR values for all 100 genes were calculated with p-values shown to be < 0.05. **B.** Normalized enrichment scores for GSEA analysis of MSigDB curated pathways / gene sets enriched in the impaired laminin adhesion cell lines with nominal *p*-values < 0.05. Bars highlighted in red are those pathways associated with cytokine signalling and inflammation.

**Figure 4:**
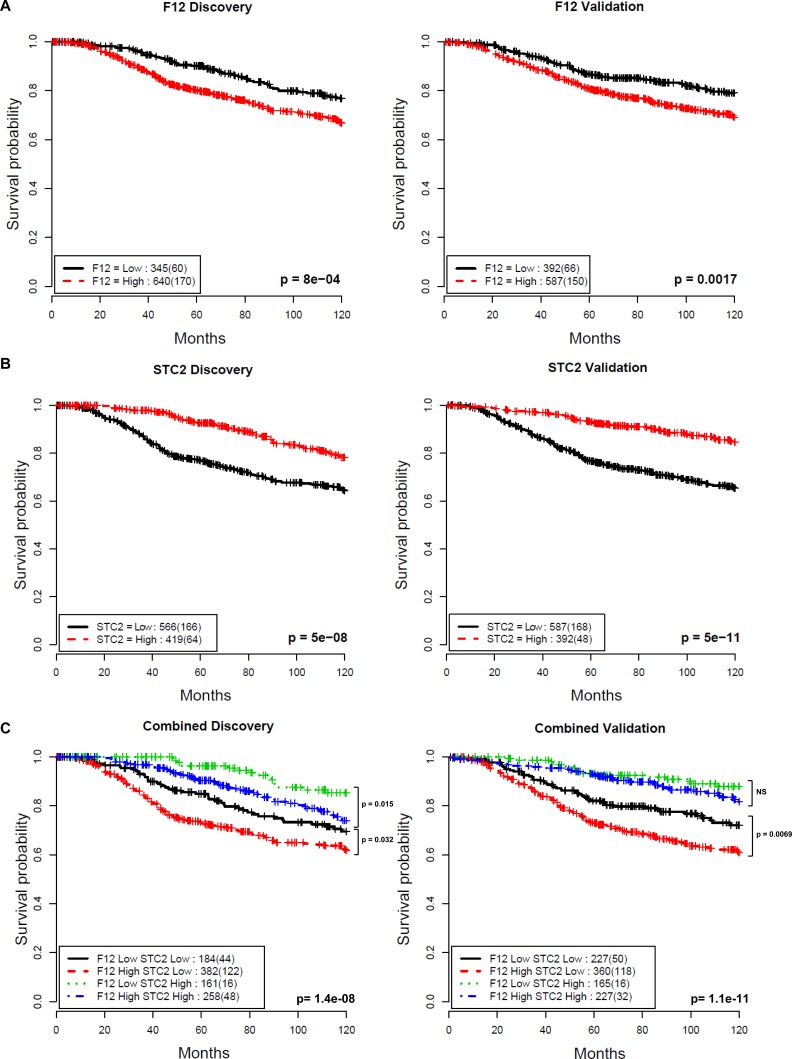
Association between F12 and STC2 gene expression and disease-specific survival in breast cancer Kaplan-Meier curves illustrate differences in disease-specific survival in patient groups in two subsets stratified by **A.** F12, **B.** STC2 and **C.** F12 + STC2 gene expression levels. The thresholds for dichotomising two indices were optimised in the discovery cohort (left) and then used without modification in the validation cohort (right) (0.2718 for F12, 0.2801 for STC2). Numbers in the legend show the number of patients in each group and numbers in brackets show the number of disease-specific deaths. Log-rank test p-values show significant differences between F12-high/STC2-low and F12-low/STC2-low groups. NS is not significant.

Unbiased analysis of the gene expression data revealed the top 50 genes, out of 21,009 genes incorporated into the analysis, that were positively and negatively correlated with impaired laminin adhesion in the cell line panel (Figure 3A). A selection of 15 genes from this signature was validated by RT-qPCR and show good concordance with the CCLE microarray data (Supplemental Table 3). Ontology analysis of genes that are upregulated in the cells with impaired laminin adhesion (AU565, HCC1954, SkBr3 and ZR75.30) using GSEA showed an enrichment of genes for cell motility (Figure 3B). Additionally, there was an unexpected enrichment of multiple gene sets associated with cytokine signalling and the inflammatory response. These included the cytokine pathway, NFκB induced, EPO NFκB pathway, Natural Killer (NK) cells pathway, Interleukin-1 receptor (IL1R), Interleukin-12 (IL12), Interleukin-10 (IL10), Interleukin-6 (IL6) and Interleukin-3 (IL3) pathways (Figure 4B). This analysis suggests that cytokine signalling may play a role in the modulation of cancer cell adhesion. Other important and well-characterised oncogenic pathways that were found to be enriched included the Epidermal Growth Factor Receptor (EGFR), Sprouty (SPRY) and Hypoxia Inducible Factor (HIF) signalling pathways.

### Impaired laminin adhesion identifies prognostic genes for breast cancer disease-specific survival

Given the important functional roles of laminin in breast cancer progression and metastasis [29], we hypothesised that genes enriched in the subset of cell lines with impaired laminin adhesion may have prognostic value for disease outcome. We focused on the top 50 genes that were either positively or negatively correlated with impaired laminin adhesion (Figure 3A). The METABRIC (Molecular Taxonomy of Breast Cancer International Consortium) study is a molecular and pathological characterisation of almost 2000 clinically annotated breast cancer specimens with long-term clinical follow-up [30]. To examine these genes and their association with 10-year breast cancer disease-specific survival, we performed univariate Cox regression analysis on the 100 genes correlated with impaired laminin adhesion in the METABRIC dataset. The optimal cut-off for dichotomizing each gene was selected using the discovery cohort (*n* = 985) and then tested in the independent validation cohort (*n* = 979).

This analysis showed that the gene F12 is a prognostic factor (discovery cohort: hazard ratio HR = 1.65, 95% confidence interval CI = 1.23 - 2.21, *p* = 8.0×10^−4^; validation cohort: HR = 1.58, CI = 1.19 - 2.12, *p* = 0.0017, Figure 4A) while the gene STC2 is a negative prognostic factor (discovery cohort: HR = 0.46, CI = 0.34 - 0.61, *p* = 5.0×10^−8^; validation cohort: HR = 0.36, CI = 0.26 - 0.49, *p* = 5.6×10^−11^, Figure 4B) for overall survival. Multivariate Cox proportional hazards analysis demonstrates that the prognostic value of these genes was independent of known prognostic variables of node status, size, grade, age, ER status and HER2 status in both cohorts (F12 discovery cohort: HR = 1.47, CI = 1.09 - 1.97, *p* = 0.012; validation cohort: HR = 1.53, CI = 1.13 - 2.08, *p* = 0.0064, STC2 discovery cohort: HR = 0.71, CI = 0.51 - 0.99, *p* = 0.043; validation cohort: HR = 0.51, CI = 0.35 - 0.75, *p* = 0.00056, Table 2).

**Table 2:** Multivariate analysis of the prognostic value of F12 or STC2 for disease-specific survival in two cohorts of breast cancer patients

Variable	Discovery Cohort (*n*=985)		Validation Cohort (*n*=979)	
	HR (95% CI)	*p*	HR (95% CI)	*p*
**F12**	1.47 (1.09 – 1.97)	0.012*	1.53 (1.13 – 2.08)	0.0064**
**Node**	2.04 (1.54 – 2.71)	7.8×10^−7^**	2.45 (1.80 – 3.34)	1.2×10^−8^**
**Size**	1.44 (1.13 – 1.84)	0.0034**	1.57 (1.23 – 2.00)	0.00029**
**Grade**	1.36 (1.05 – 1.75)	0.018*	1.31 (1.00 – 1.70)	0.048*
**HER2**	1.63 (1.17 – 2.28)	0.0042**	1.28 (0.90 – 1.83)	0.17
**ER**	0.63 (0.45 – 0.89)	0.0084**	0.55 (0.39 – 0.77)	0.00049**
**Age**	1 (0.99 – 1.01)	0.68	1.01 (1.00 – 1.02)	0.14
**STC2**	0.71 (0.51 - 0.99)	0.043*	0.51 (0.35 – 0.75)	0.00056**
**Node**	2.03 (1.53 - 2.70)	1.1×10^−6^**	2.43 (1.79 - 3.31)	1.5×10^−8^**
**Size**	1.44 (1.13 - 1.85)	0.0036**	1.57 (1.23 – 2.00)	0.00025**
**Grade**	1.31 (1.01 -1.70)	0.039*	1.23 (0.95 – 1.60)	0.12
**HER2**	1.63 (1.17 – 2.27)	0.0041**	1.29 (0.91 – 1.82)	0.16
**ER**	0.73 (0.51 – 1.03)	0.071*	0.72 (0.51 – 1.03)	0.07
**Age**	1 (0.99 – 1.01)	0.56	1.01 (1.00 – 1.02)	0.11

* ***p***<0.05,

** ***p***<0.01

Combining F12 and STC2 stratified patients into 4 subgroups (F12-high/STC2-low, F12-low/STC2-high, F12-high/STC2-high and F12-low/STC2-low) with F12-low/STC2-high being associated with favourable outcome (10-year survival probability of 90.0% discovery, 90.3% validation) and F12-high/STC2-low patients having a much poorer prognosis (10-year survival probability of 68% discovery, 67.2% validation) (Figure 4C). F12 expression levels were able to further stratify the STC2-low group into 2 subgroups with significantly different outcomes (discovery cohort: HR = 1.45, CI = 1.03-2.05, *p* = 0.032; validation cohort: HR = 1.57, CI = 1.12 - 2.18, *p* = 0.0069) (Figure 4C). Despite the gene expression signature being derived from a HER2+ panel of cell lines, we found no subtype specificity for these two genes across HER2, Luminal A, Luminal B and Basal breast cancer subtypes in the METABRIC cohort (data not shown), indicating the general applicability of the *in vitro* cell adhesion phenotypic screen to discover prognostic factors independent of subtypes.

## DISCUSSION

Cell-ECM adhesion is a fundamental process that is important in multiple hallmarks of breast cancer, including conferring a proliferative advantage, inducing pro-survival pathways and driving cell migration and invasion [31, 32]. In this study, we employed a tumour cell-ECM interaction screen to systematically assess the cell adhesion properties in a panel of breast cancer cell lines across six different ECM components. This led to the identification of a subset of cell lines which exhibit impaired adhesion to laminin. Laminin is a major component of the BM which acts as a physical barrier that is degraded to facilitate tumour invasion. Immunohistochemistry studies on primary and metastatic breast cancer specimens have found that laminin positivity itself has no prognostic significance for disease-free and overall survival [33–35]. We show that the F12 and STC2 genes derived from a gene expression signature of impaired laminin adhesion are independent prognostic factors for overall survival in breast cancer. Specifically, patients with the F12-high/STC2-low profile found in the cells with impaired laminin adhesion, had a poorer outcome compared to those with F12-low/STC2-high gene expression. Cell migration is a key determinant of cancer cell dissemination and metastasis and it has been shown that increased cell adhesion to laminin inhibits breast cancer cell migration [36–40]. In keeping with this model, the ontology analysis performed in this study showed that there is an enrichment of genes for cell motility in breast cancer cells with impaired laminin adhesion. Given that our data demonstrates that patients with a F12-high/STC-low profile have a poorer outcome, a possible hypothesis is that poor breast cancer disease-specific survival may be the result of reduced laminin adhesion and increased tumour cell migration, leading to elevated cancer cell dissemination from the primary site.

Despite our data demonstrating that cell lines with impaired laminin adhesion were enriched in genes associated with cell motility, there was no correlation of laminin receptor mRNA expression levels with cell adhesion to laminin (Table 1). Laminins are a class of heterotrimeric proteins composed of α, β and γ chains which are predominantly found in the basement membrane that separates the breast epithelial from the stroma [41, 42]. Breast cancer cells interact with laminins via the integrins which are heterodimeric receptors comprising an α and β subunit. Multiple integrins are known to bind to laminins including the α3β1, α6β1, α7β1 and α6β4 receptors [27, 43]. The collagen binding α1β1, α2β1 and α10β1 integrins have also been shown to bind to laminins [27]. The binding of integrins to laminins triggers the formation of a multi-protein signalling complex called the adhesome [44]. This dynamic process is known as outside-in signalling and involves the recruitment of integrin-binding and actin-binding proteins as well as kinases [45]. Prominent pathways that are activated downstream of integrin binding are the FAK, PI3K, ERK and RhoA pathways which drive a range of pathological functions including tumour cell migration and invasion [46, 47]. Additionally, several studies report significant crosstalk between α6β1 and α6β4 integrins and HER2 in breast cancer cell lines [48, 49]. Our data shows no correlation between integrin mRNA levels and laminin adhesion which is in agreement with previous reports indicating that steady state integrin RNA expression levels are poor predictors of the cell adhesion phenotype [20]. Given that cell adhesion is mediated by specific integrin heterodimers, it is likely that future assessment of cell surface levels of integrins as measured by flow cytometry will be more predictive of ECM-cell adhesion capacity in our assays [50].

STC2 encodes for the stanniocalcin 2 protein which is a glycoprotein hormone that is decreased in cell lines with impaired laminin adhesion. This protein was first identified in bony fish and has since been shown to be ubiquitously expressed in almost all mammalian tissues where it plays a role in the regulation of calcium and phosphate transport [51]. In agreement with the poorer outcome associated with low STC2 expression presented in this study, prior reports have shown that reduced stanniocalcin 2 expression promotes breast cancer cell proliferation, migration and invasion. Stanniocalcin 2 expression is regulated by hormone signalling and an increase in stanniocalcin 2 expression levels leads to a reduction in breast cancer cell viability *in vitro* [52]. Overexpression of stanniocalcin 2 also suppressed breast cancer cell migration and inhibited tumorigenesis and metastasis in a xenograft model of breast cancer [53]. Consistent with our findings, in a previous study STC2 levels were analysed in 110 breast cancer cases and found to be associated with favourable prognosis in hormone receptor (HR)-positive breast cancer patients treated with adjuvant hormone therapy [54]. Similarly, another study showed in a panel of 245 breast tumours that STC2 positivity was prognostic for favourable disease-free survival [55]. Larger cohorts comprising defined stages and subtypes of breast cancer will be necessary to evaluate the prognostic potential of STC2, especially in the context of HR-positive breast cancer.

The F12 gene encodes for the plasma coagulation factor XII (FXII) which is a serine protease precursor that plays a role in the normal blood clotting process [56]. In addition to its role in blood clotting, FXII functions as a potent mitogen through the binding of the urokinase plasminogen activator receptor (uPAR) [57–59]. This leads to the activation of both the ERK1/2 and AKT pathways which promotes cell proliferation [57, 60]. Interestingly blockade of integrins β1, α3 and α5 inhibited FXII induced signalling suggesting crosstalk between the uPAR and laminin and fibronectin receptor pathways [60, 61]. It is still unclear if FXII is a *bona fide* cancer driver but it shares remarkable similarities with the activation of uPAR by urokinase plasminogen activator (uPA), an important driver of epithelial-mesenchymal transition in breast cancer [62, 63]. To our knowledge, there have been no clinicopathological studies performed to evaluate the prognostic value of F12 and our study is the first to demonstrate a novel association between high F12 gene expression levels and unfavourable prognosis in breast cancer. Our findings are consistent with the function of FXII as a mitogen and warrants further study of its potential role as a candidate oncogene in breast cancer.

Analysis of molecular pathways associated with impaired laminin adhesion shows an enrichment of gene sets for cytokine signalling and inflammation. Previous reports indicate that cytokines have the capacity to modulate ECM adhesion in immune cells [64–67]. For instance, pro-inflammatory cytokines TNFα, IFNα and INFγ promote laminin adhesion while TGFβ1 had the opposing effect in microglial cells [65]. Adhesion of CD4+ T cells to laminin has been shown to increase in response to TNFα [66]. Pro- and anti-adhesive cytokine activities are less well studied in cancer cells. Treatment of colon carcinoma HT-29 cells with IL-4 and TNFα led to an increase in fibronectin adhesion *in vitro* with a corresponding decrease in lung colonizing potential *in vivo* [68]. In the MIA PaCa2 pancreatic cancer cell line, IL-1β reduced adhesion to laminin while IL-1α increased fibronectin adhesion but impaired collagen I adhesion [69]. In another study, both IL-1β and TNFα promoted MG-63 osteosarcoma cell adhesion on laminin [70]. Our data documents a strong association between cytokine signalling, inflammation and the regulation of laminin adhesion in breast cancer cells. In view of the increasing appreciation of the role of inflammation in driving breast cancer progression and the potential for immunotherapies in this disease [71], future experimental analysis of the impact of cytokines in modulating breast cancer cell adhesion will be required to establish if this is a major contributor to tumour development.

It should be noted that gene expression profiles from the METABRIC dataset are derived from clinical specimens composed of a heterogeneous population of different cell types, including tumour and stromal cells [72]. There may also be considerable intra-tumoural heterogeneity within tumour cell populations which is masked in population-level gene expression measurements [73]. In contrast, the expression profiles from the CCLE compendium are generated from breast cancer cell lines which are largely homogenous in nature. While we have shown that the F12/STC2 gene pair is an independent prognostic factor for breast cancer survival, given the heterogeneity inherent in clinical specimens we cannot exclude the possibility that alterations in F12 and STC2 levels may be contributed in part by stromal cells. Future work using single-cell RNA-sequencing methods may shed light on the relative contributions of tumour versus stromal cells in breast cancer prognostication [74].

One limitation of our study is that all adhesion measurements were performed on ECM coated surfaces as opposed to 3-dimensional (3D) culture systems. It is recognised that cells embedded in 3D ECM cultures better simulate the tumour microenvironment *in vivo* by recapitulating features such as hypoxic gradients [75, 76]. There may be context-dependent signalling differences in 3D versus 2-dimensional (2D) cultures where cells in 3D ECM conditions may be exposed to spatial cues and tensional forces necessary to trigger integrin bidirectional signalling [76]. Notwithstanding the limitations of the simplified experimental setup of ECM coated surfaces, our study demonstrates that novel prognostic factors can still be readily identified with this approach. We anticipate that the use of more physiologically relevant models such as 3D ECM cultures is likely to lead to the identification of additional independent adhesion-associated prognosticators of breast cancer survival.

In summary, we show for the first time that the systematic analysis of cell-ECM interactions has utility in defining new prognostic factors for breast cancer disease-specific survival. Our study also sheds light on a potential role of cytokine signalling in the regulation of cancer cell adhesion. Future analysis in a larger panel of cell lines with additional ECM substrates will ascertain the general applicability of this approach in the detection of prognostic biomarkers in other cancer types.

## MATERIALS AND METHODS

### Cell culture

Breast cancer cell lines were obtained from ATCC (Manassas, VI, USA). BT474, SkBr3, UACC812 and JIMT1 were grown in Dulbecco's Modified Eagle's Medium (DMEM) supplemented with 10% foetal bovine serum (FBS). ZR75.30, AU565 and HCC1954 were grown in RPMI-1640 supplemented with 10% foetal bovine serum. All cells were cultured in a 37°C incubator with 5% CO_2_.

### Extracellular matrix coating

Collagen I (rat tail; Sigma Aldrich) and collagen IV (from human placenta; Sigma Aldrich) were diluted to 20ug/mL in 0.5M acetic acid, tenascin C (human purified; Millipore) was diluted to 5ug/mL in PBS, fibronectin (from human plasma; Millipore), laminin (mouse purified; Millipore) and hyaluronan (high molecular weight; R&D Systems) were diluted to 20ug/mL in PBS. Diluted coatings were applied to cover wells or flasks for 2h at room temperature, then removed and washed with PBS. PBS was removed and cells were seeded in their appropriate media.

For assays using pairwise ECM combinations, collagen I and collagen IV were diluted to 40ug/mL in 0.5M acetic acid, tenascin C was diluted to 10ug/mL in PBS, and other ECM components were diluted to 40ug/mL in PBS. These ECM dilutions were mixed 1:1 for each pairwise combination prior to plating. Additional concentration-matched single ECM controls were generated by further diluting single ECM components 1:1 in PBS.

### Cell adhesion and proliferation assays

For 72 hour assays, cells were seeded at 2-5×10^3^ per well (as appropriate per cell line) onto coated or uncoated 96-well CellCarrier imaging plates (Perkin Elmer). Harvest was 72h post-treatment, and 8h (HCC1954, SkBr3, JIMT1, AU565) or 24h (BT474, ZR75.30, UACC812) prior to harvest, 10uM BrdU (Sigma Aldrich) was added to media. To harvest, cells were fixed in 4% formaldehyde for 15 minutes, then washed 2x with PBS and permeabilised with 0.25% Triton X-100 in PBS for 10 minutes, washed 2x then blocked with 10% FBS in PBS. 50uL primary anti-BrdU antibody (BU20a; Affymetrix) diluted 1:1000 and RQ1 DNAse (Promega) diluted 1:200 in DNAse buffer (40mM Tris-HCl (pH 8), 10mM MgSO_4_, 1mM CaCl_2_) was added to each well and incubated at 37°C for 3h. Wells were washed 2x TPBS, then secondary AlexaFluor 488 goat anti-mouse antibody (Life Technologies) and Hoechst 33342 (Tocris) were added 1:1000 and 1:2000 respectively in 10% FBS in PBS for 1h. Plates were washed 2x in TPBS, then plates were imaged on an Operetta high-content imager (Perkin Elmer) using a 10x High NA lens. Images were analysed using Harmony software (Perkin Elmer), and BrdU positivity was defined as number of Hoechst-stained nuclei also showing 488 stain positivity > 2 times background staining.

### Gene expression analysis

We obtained the microarray gene expression data from the Cancer Cell Line Encyclopaedia (GSE36133). These cell lines were profiled using Affymetrix HG-U133 Plus 2.0 microarrays. Raw CEL files for these cell lines were normalized using Robust Multiarray Average (RMA) approach in Affymetrix Power Tools (APT). Gene Set Enrichment Analysis (GSEA) software version 2.2.2 from the Broad Institute [77] was used to identify genes and pathways enriched in cell lines with impaired laminin adhesion. Our input file contained expression data for 21,010 genes and 7 cell lines. We used 1000 gene set permutations for the analysis and pathways with nominal *P* values < 0.05 were considered significant. We used the 522 pathways in the curated gene sets (C2.V1) collection from MSigDB. Gene sets with less than 10 genes were excluded from our analysis. GSEA software was also used to identify 50 genes with the strongest positive correlations and 50 genes with the strongest negative correlations to the impaired laminin adhesion phenotype. Statistical significance of gene correlations to the impaired laminin adhesion phenotype was determined using the Holm-Sidak method which corrects for multiple comparisons using Graphpad Prism software. *P* < 0.05 was deemed significant.

Gene expression data of 17,653 genes from the METABRIC study were profiled using Illumina HT-12 v3 microarray. The top 50 genes that were positively and negatively correlated with impaired laminin adhesion in the cell line panel were subjected to further analysis. As outlined in the METABRIC study, the discovery and validation cohorts were defined by hospital sites, for further details of clinical specimens please refer to [30]. Survival analysis was performed with breast-cancer-specific 10-year survival data. The Kaplan-Meier estimator was used, and the log-rank test was performed to test differences among groups. For univariate and multivariate analysis, the Cox proportional hazards regression model was fitted, and 95% confidence intervals computed to determine prognostic values; log-rank test *p* < 0.05 was considered significant. In the discovery set, a search for optimal threshold for dichotomizing gene expression data was carried out by searching stepwise from 40 to 60 percentiles at an interval of 5. The cut-offs that displayed the highest prognostic significance with log-rank test were selected and used for the validation cohort. Multivariate survival analysis was carried out in both patient cohorts using gene expression data, node status, ER status, HER2 status, age, tumour size and grade.

### Real-time quantitative PCR

Total RNA was isolated from cultured cell lines using Trizol reagent (Life Technologies), treated with DNase I (Promega) and reverse transcribed into cDNA with SuperScript III First-strand Synthesis kit (Life Technologies). qPCR was carried out using SYBR green PCR master mix (Life Technologies) on an Applied Biosystems 7900HT instrument. Data were normalized against β-actin levels and analysed using Applied Biosystems RQ manager software. The gene-specific primers used are listed in Supplemental Table 4.

## SUPPLEMENTARY MATERIALS FIGURES AND TABLES


