# Supplementary material for: Systematic analysis of tumour cell-extracellular matrix adhesion identifies independent prognostic factors in breast cancer
Source: Oncotarget. 2016 Aug 17;7(39):62939–53. doi: 10.18632/oncotarget.11307 (PMC5325338; doi:10.18632/oncotarget.11307)
Supplement: Supplementary file 1 [file oncotarget-07-62939-s001.pdf]

Systematic analysis of tumour cell-extracellular matrix adhesion identifies independent prognostic factors in breast cancer

Supplementary Material

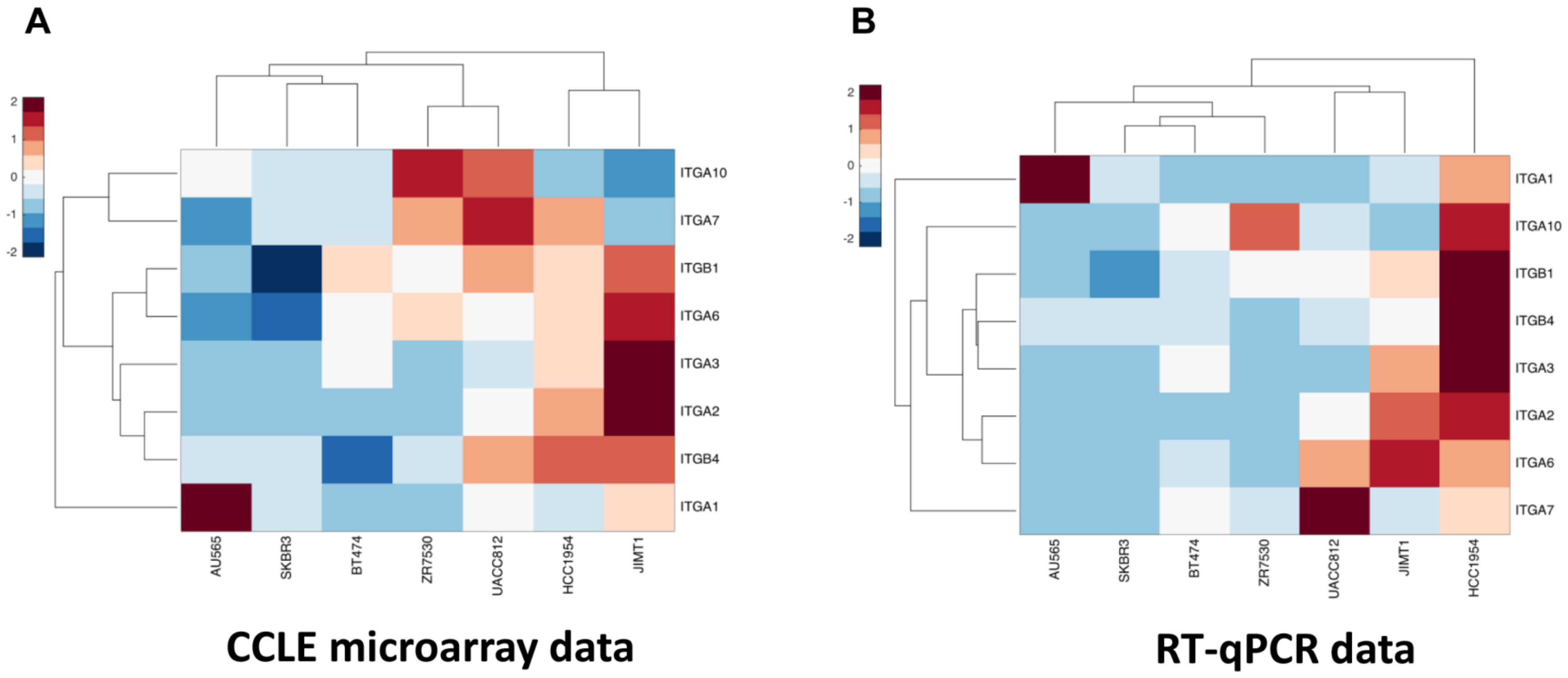

Supplemental Figure 1: Two way clustering of laminin binding integrin mRNA levels. (A) CCLE microarray data and (B) RT-qPCR measurements.

Supplemental Table 1: Laminin binding integrin gene expression levels as measured by microarray analysis (CCLE) and RT-qPCR. Student's t-test compares the statistical difference in integrin gene expression levels between the impaired laminin adhesion and preserved laminin adhesion cells.

CCLE microarray data

	Impaired laminin adhesion				Preserved laminin adhesion			
**Integrins**	**AU565**	**HCC1954**	**SKBR3**	**ZR7530**	**BT474**	**JIMT1**	**UACC812**	**t-test**
ITGA1	5.78	3.81	3.80	3.76	3.69	4.41	4.22	0.76
ITGA2	7.31	9.41	7.21	7.34	7.09	10.53	8.41	0.50
ITGA3	5.91	7.89	5.80	5.94	7.21	10.14	6.18	0.35
ITGA6	4.88	8.50	3.55	7.66	7.49	10.94	7.33	0.20
ITGA7	4.37	4.80	4.53	4.84	4.55	4.52	5.04	0.75
ITGA10	5.69	5.32	5.36	6.56	5.36	5.12	6.34	0.81
ITGB1	11.84	12.55	11.17	12.52	12.57	13.22	12.92	0.07
ITGB3	4.98	4.49	4.64	4.93	4.85	4.70	4.71	0.97
ITGB4	7.44	9.40	7.51	7.51	6.30	9.66	8.85	0.80

RT-qPCR data

	Impaired laminin adhesion				Preserved laminin adhesion			
**Integrins**	**AU565**	**HCC1954**	**SKBR3**	**ZR7530**	**BT474**	**JIMT1**	**UACC812**	**t-test**
ITGA1	69511.13	36561.31	12337.31	0.30	1.00	9196.39	14.89	0.18
ITGA2	1.03	8.31	1.29	1.04	1.00	7.79	3.55	0.68
ITGA3	0.05	4.27	0.07	0.19	1.00	2.80	0.20	0.89
ITGA6	0.01	3.67	0.00	0.55	1.00	4.59	3.13	0.24
ITGA7	0.29	1.83	0.15	0.57	1.00	0.59	4.75	0.40
ITGA10	0.45	2.42	0.19	2.19	1.00	0.48	0.69	0.39
ITGB1	0.57	3.47	0.37	1.39	1.00	2.04	1.68	0.88
ITGB3	0.45	28573.57	32.02	19.53	1.00	11.47	125.93	0.39
ITGB4	0.83	10.64	0.70	0.35	1.00	2.87	1.63	0.65

Supplemental Table 2: Correlation of mRNA levels (measured by RT-qPCR) of laminin integrins and cell adhesion.
*** Statistical significance of correlation where p<0.05.**

	Receptor	R (Correlation coefficient)	P-value
Laminin	ITGA1	-0.25	0.59
	ITGA2	0.18	0.69
	ITGA3	0.08	0.87
	ITGA6	0.47	0.29
	ITGA7	0.44	0.32
	ITGA10	-0.42	0.35
	ITGB1	0.04	0.93
	ITGB4	-0.13	0.78

Supplemental Table 3: Spearman's correlation analysis of CCLE microarray data and RT-qPCR measurements

**Gene**	**Spearman ρ**	**p value**
CRISP3	0.9643	0.0028
PROM1	0.9643	0.0028
KRT23	0.8571	0.0238
WNT5A	0.8214	0.0341
VTCN1	0.8571	0.0238
ZNF750	0.8214	0.0341
HPSE	0.8571	0.0238
MDK	0.9643	0.0028
ZNF415	0.8929	0.0123
CBFA2T3	0.8571	0.0238
ZSCAN18	1	0.0004
ZNF257	0.9643	0.0028
LONRF2	0.8571	0.0238
ZNF238	0.9643	0.0028
HSPB8	0.8214	0.0341

Supplemental Table 4: Gene-specific primers used in qPCR

Gene	Forward primer (5'-3')	Reverse primer (5'-3')
Actin B	GACAGGATGCAGAAGGAGATCACT	TGATCCACATCTGCTGGAAGGT
CRISP3	TACAGACACAGTAACCCAAAGGA	TGGATTGCTTGTGACCATGAG
PROM1	GGCCCAGTACAACACTACCAA	ATTCCGCCTCCTAGCACTGAA
KRT23	TACTAGGCGGAAATGGGAAGG	TCTTACCATCCACTATCTGCTCC
WNT5A	ATTCTTGGTGGTCGCTAGGTA	CGCCTTCTCCGATGTACTGC
VTCN1	TTTAAGGCCAATACACGGGAGC	ACACCTTCCTTCAGCCATTGT
ZNF750	CACTGGCTTTACACAACCCCA	GGTAAGGCGAGTAGATGGTGG
HPSE	TCATCAATGGGTCGCAGTTAGG	TTAGCCGTCTTTCTTCGAGGC
MDK	ATGTGACCGGCTCAGACC	GCCGCCCTTCTTCACCTTAT
ZNF415	TGGATCTGTCTCGTAACTGTGT	TGATTTCCCTGAAGCAAAACTCT
CBFA2T3	CACTCACCAACAGCCATCAAT	CGTCAATGTCGAGTTCACCAG
ZSCAN18	GCGGGCTCATCCTCAATTCTT	CCTCTTCGGTCTTTGCTTCTC
ZNF257	GTCTTCCCTGGTCTGTGTCC	TCTGGGCAAAGGTCTTCAGC
LONRF2	AACTTTCGGAATTATTGGCAAGC	CGTCTCTGGTCAGATTTGACAGT
ZNF238	CCACCTCTTTTACAAGGACCAG	CTAGCACGTCTTCAATGGGC
HSPB8	AAATGTTAGAGGGTGCGGGG	GCCAATTGCGCTATCCTGTG
ITGA1	GCTCCTCACTGTTGTTCTACG	CGGGCCGCTGAAAGTCATT
ITGA10	GGGAATCAGTATTACACAACGGG	CCACAACATCTATGAGGGAAGGG
ITGA2	TGTGGCTTGGAGTGACTGTG	TCATTGCCTCGCACGTAGC
ITGA3	GGCGGTGTTATGTCCTGAGTC	AATCGCCCATCACAAAAGCTC
ITGA6	TATTGACTCGGGGAAAGGTCT	CCAGCCATCACTGTTGAGG
ITGA7	AACATCACCCACGCCTATTCC	GTTGGTAGTCACCTAAGTGGC
ITGB1	CAAGAGAGCTGAAGACTATCCCA	TGAAGTCCGAAGTAATCCTCCT
ITGB4	GCTTCACACCTATTTCCCTGTC	GACCCAGTCCTCGTCTTCTG
